# Extracellular Vesicles From Adipose Tissue-Derived Stem Cells Affect Notch-miR148a-3p Axis to Regulate Polarization of Macrophages and Alleviate Sepsis in Mice

**DOI:** 10.3389/fimmu.2020.01391

**Published:** 2020-07-03

**Authors:** Xiaozhi Bai, Junjie Li, Lincheng Li, Mingchuan Liu, Yang Liu, Mengyuan Cao, Ke Tao, Songtao Xie, Dahai Hu

**Affiliations:** ^1^Department of Burns and Cutaneous Surgery, Xijing Hospital, Fourth Military Medical University, Xi'an, China; ^2^Emergency Department, Xijing Hospital, Fourth Military Medical University, Xi'an, China; ^3^Brigade 4, College of Basic Medicine, Fourth Military Medical University, Xi'an, China; ^4^Chinese People's Liberation Army Hospital 961, Qiqihar, China

**Keywords:** ADSCs, extracellular vesicles, inflammation, notch, microRNA-148a-3p

## Abstract

Extracellular vesicles (EVs) from adipose tissue-derived stem cells have been reported to attenuate lipopolysaccharide (LPS) induced inflammation and sepsis while the specific mechanism is unclear. This study explored the underlying molecular mechanisms of EVs from adipose tissue-derived stem cells in reducing inflammation. LPS- induced macrophage models and mice model were established to mimic inflammation *in vitro* and *in vivo*. EVs were extracted from adipose tissue-derived stem cells and identified. It was found that proinflammatory cytokines, including IL-1β, IL-6, and TNF-α, substantially decreased after EVs were applied to LPS-stimulated macrophages and mice, and thus, LPS induced M1 polarization was inhibited and sepsis was strongly alleviated. In the LPS induced macrophages, the expression of Notch signaling molecules and the activation of the NF-κB pathway were substantially decreased after the administration of EVs. Then, *RBP-J*^−/−^ mice and macrophages were used. It was found that the miR-148a-3p level was significantly lower in the *RBP-J*^−/−^ macrophages than in the wildtype macrophages. In the LPS induced macrophages, the increasing of miR-148a-3p was milder in the *RBP-J*^−/−^ macrophages than in the wild type macrophages. Then, miR-148a-3p was overexpressed in macrophages and mice, and we found that the expression of proinflammatory cytokines was increased both *in vivo* and *in vitro*. The protective effect of EVs in LPS induced sepsis was diminished by the overexpression of miR-148a-3p. In conclusion, we proved that EVs could attenuate inflammation and further protect organ function by regulating the Notch-miR148a-3p signaling axis and then decreasing macrophage polarization to M1.

## Introduction

Sepsis is a life-threatening syndrome caused by dysregulated host-infection response, which leads to multiple organ dysfunction syndrome (MODS) and has a nearly 40% rate of mortality (1, 2). It has been reported that increased systemic levels of endotoxins during sepsis are responsible for the activation of innate immune cells such as macrophages, leading to the breakout of inflammatory mediators and cytokines (3). Macrophages participate in not only xenobiotic-induced inflammation but also endogenous inflammation, such as aseptic inflammatory response (4, 5). Macrophages can be broadly polarized to two categories: the classically activated proinflammatory type M1 and the alternatively activated restorative type M2. M1 macrophages are antigen-presenting cells that can be induced by LPS or IFN-α. They are mainly responsible for producing proinflammatory mediators such as tumor necrosis factor-α (TNF-α), interleukin 6 (IL-6), interleukin 1 (IL-1), monocyte chemoattractant protein-1 (MCP-1), and so on (6, 7). These proinflammatory cytokines may lead to the cascade of other inflammatory cytokines and aggravate tissue damage.

It has been reported that mesenchymal stem cells (MSCs) can alleviate inflammation during sepsis and then increase the survival rate (8, 9). Adipose tissue-derived stem cells (ADSCs), a class of mesenchymal stem cells derived from adipose tissue, can attenuate inflammation by paracrine secretion of various cytokines (10, 11). Numerous reports have confirmed that paracrine may be one of the main mechanisms underlying the curative effects of ADSCs (12). In particular, EVs, including exosomes and microvesicles, can transfer from cell to cell, are promising molecules both in research and clinical applications (13).

EVs are lipid nanovesicles secreted by multiple cell types, including stem cells, immune cells, cancer cells and neurons (14, 15). Recent studies have shown that EVs participate in intercellular communication, which are essential in various physiological processes, including tissue homeostasis, immune response and anti-inflammatory functions (16). EVs can communicate and transfer information by secreting multiple cytokines, chemokines, nucleic acids, and growth factors, controlling the expansion and progression of diseases (17). They are released and spread to adjacent cells to enable cell-cell communication (18). It has been reported that ADSC-derived exosomes are involved in the anti-inflammatory activity of ADSCs (19). The clinical use of stem cells is limited by the poor survival rate after transplantation, and explorations of EVs including exosomes are promising. However, the underlying mechanism by which ADSC derived EVs attenuate inflammation is still unclear.

In this study, we explored the underlying protective mechanisms of ADSC derived EVs (ADSC-EVs) in lipopolysaccharide (LPS)-induced inflammation and found that Notch signaling and downstream microRNA-148a-3p are essential for ADSC-EVs to reduce inflammation. Collectively, our study provides a promising possible therapeutic strategy for inflammation in sepsis.

## Materials and Methods

### Animals

All the experimental protocols in this study followed the guidelines approved by the Ethics Committee of Xijing Hospital affiliated with the Fourth Military Medical University (No: XJYYLL-2015206). Healthy male adult wild-type (WT) mice and myeloid-specific *RBP-J*-deficient mice (*RBP-J*^−/−^mice) weighing 20 to 25 grams were included in this study. Mice ~6–8 weeks old were provided by the Experimental Animal Center of The Fourth Military Medical University (FMMU, Xi'an, China). All mice were fed a standard diet and water *ad libitum*. Mice were anesthetized with isoflurane inhalation. We ensured that experienced researchers conducted the invasive procedure in a sterile environment using sterilized instruments. In short, we tried our best to minimize the pain of the mice.

### Isolation and Culture of ADSCs

ADSCs were isolated using mechanical and enzymatic means described by Zhang et al. (20). ADSCs were obtained from the subcutaneous adipose tissues of the inguinal area of C57B6/L mice. The adipose tissues were washed with phosphate-buffered saline (PBS) to remove most erythrocytes. Then, the tissue was fragmented and digested with 1 mg/ml type I collagenase for 40 min at 37°C on a shaker. Subsequently, the digested tissue was filtered through a 100 μm cell strainer, centrifuged at 400 g for 5 min and washed twice with PBS. The cell pellet was then suspended in Dulbecco's modified Eagle's medium (DMEM, Gibco, US) with 1% bovine serum albumin (BSA), treated with erythrocyte lysis buffer and then centrifuged again at 500 g for 7 min at 4°C. Then, the cells were cultured in DMEM/F12 (Gibco, US) with 10% FBS (Excell Bio, China), 1% (v/v) penicillin/streptomycin and 2 mM glutamine in a 5% CO_2_ incubator.

### Identification of ADSCs

ADSCs were labeled with fluorescence-conjugated antibodies or isotype control for 30 min. The antibodies used in flow cytometry were listed in Table 1. For osteogenic differentiation, cells were incubated with osteogenic medium, which contains 10% FBS, 10 mM β-glycerophosphate, 50 mg/L L-ascorbic and 10^−7^ M dexamethasone in DMEM/F12 medium, for 2 weeks and then evaluated by Alizarin Red S staining for 5 min. For adipogenic differentiation, cells were incubated with adipogenic medium, which contains 10% FBS, 1 μM dexamethasone, 10 μM insulin, 20 μM indomethacin and 0.5 mM 3-isobutyl-1-methyl xanthine (IBMX) in DMEM/F12 medium, for 2 weeks and then assessed using Oil red O staining for 30 min. Then, cells were washed with PBS, and images were taken by optical microscope.

**Table 1:** Antibodies used for flow cytometry.

	**Antibody**	**Dilution Ratio**	**Company**
CD31	APC Rat Anti-Mouse CD31	1:800	Becton-Dickinson, US
	APC Rat IgG2a κ Isotype Control	1:800	
CD29	PE Hamster Anti-Mouse CD29	1:1000	
	PE Hamster IgG2, λ1 Isotype Control	1:1000	
CD34	FITC Rat anti-Mouse CD34	1:800	
	FITC Rat IgG2a, κ Isotype Control	1:800	
CD105	PE-CF594 Rat Anti-Mouse CD105	1:1000	
	PE-CF594 Rat IgG2a, κ Isotype Control	1:1000	

### EVs Isolation and Identification

EVs were isolated and purified from the supernatant of ADSCs. ADSCs were cultured in serum-free medium supplemented with 10% FBS and 1% penicillin/streptomycin at 37°C in a 5% CO_2_ incubator for 48 h. FBS was ultracentrifuged for 16 h at 120,000 g and 4°C to deplete the exosomes. Next, EVs were isolated using ultracentrifugation as described by Li et al. (21). Briefly, cell culture medium was centrifuged at 300 g for 10 min, 2000 g for 10 min, and 10,000 g for 30 min at 4°C to remove cell debris. Then, EVs were collected by centrifugation at 100,000 g for 70 min after discarding the supernatant. EVs were resuspended and washed in PBS and then centrifuged at 100,000 g for 70 min. Finally, EVs were resuspended in 400 μl of PBS and stored at −80°C. The ultrastructure and size EVs were analyzed by transmission electron microscopy (TEM) and the Zeta View® system (Particle Metrix, Germany), respectively. Four fields were randomly selected, and eight vesicles in each field were measured. The surface protein markers CD9, CD63, and CD81 and cytosolic Alix were detected by western blotting.

### Cell Culture, Grouping and Treatment

RAW264.7 cells (murine macrophage cell line) were purchased from the American Type Collection Culture (ATCC, USA). Peritoneal macrophages from WT mice and *RBP-J*^−/−^ mice were collected and incubated as described in other literature (22). Cells were cultured in RPMI 1640 medium (Gibco, US) containing 10% FBS, 100 U/ml penicillin and 100 mg/ml streptomycin and then maintained at 37°C in a humidified incubator with 5% CO_2_. The medium was changed every 2 days, and cells between the third and fifth passages were used in our experiments. RAW264.7 cells were seeded in 6-well plates, grown to 60–80% confluence, serum-starved for 12 h, and divided into five groups: Control (PBS), LPS (1 μg/ml), LPS+ 25 μg/ml ADSC-EVs, LPS+ 50 μg/ml ADSC-EVs and LPS+ 100 μg/ml ADSC-EVs. The cells were collected 4 h later for RT-PCR and 24 h later for western blotting.

### Animal Model and ADSC-EVs Administration

Healthy adult male C57B6/L mice weighing 20–25 grams were included in this study. Mice were anesthetized with 1% sodium pentobarbital and received an intraperitoneal injection of 10 mg/kg body weight LPS. Mice were randomized into LPS group and LPS+ADSC-EVs group. The LPS+ADSC-EVs group was administered 200 μg of ADSC-EVs suspended in a total of 200 μl of PBS via tail vein injection after LPS injection. The LPS group was treated with 200 μl of PBS simultaneously. Twenty-four hours after injection, mice were sacrificed.

For overexpression of miR-148a-3p in mice, miR-148a-3p agomiR (RiboBio, China) was injected into the tail vein on 3 consecutive days (12.1 μmol/kg/day). Twenty-four hours after the last injection, LPS was injected. Then, another 24 h later, mice were sacrificed. The level of miR-148a-3p in the pulmonary of mice were detected 12 h after the last injection of miR-148a-3p agomiR (Supplementary Figure 1B).

### Cell Transfection and Grouping

RAW264.7 cells were randomly divided into NC (miR mimic) group, miR mimics group, NC (miR inhibitor) group, miR inhibitor group, LPS + NC (miR mimic) group, LPS + NC (miR inhibitor) group, LPS+ miR inhibitor group and LPS + miR mimics. The miR-148a-3p mimic, miR-148a-3p inhibitor and their negative controls were transfected into RAW264.7 macrophages using Lipofectamine 2000 Transfection Reagent (Invitrogen, USA) according to the manufacturer's instructions. Twenty-four hours after transfection, cells were used for further experiments. MiR-148a-3p mimic and its negative control miR-NC, miR-148a-3p inhibitor and its negative control miR-NC were purchased from Shanghai GenePharma Co. The cells were collected 4 h later for LPS (1 μg/mL) stimulation for RT-PCR and 24 h for western blotting.

### Real-Time PCR Analysis

Total RNA was extracted from cultured macrophages using Trizol reagent (Invitrogen, Inc., CA) according to the products' instructions. RNA was reversely transcribed into cDNA using a Prime Script™ RT Reagent kit (TaKaRa, Japan). The obtained cDNA was then amplified using a RT-PCR system (Bio-Rad, US) with a SYBR™ Premix Ex Taq™ kit (TaKaRa, Japan), with GAPDH and U6 RNA (for miRNAs) as the reference genes. The relative expression was calculated using the 2–ΔΔCT method to analyze the results. (For U6 miRNA: 5′-GTGCTCGCTTCGGCAGCACATAT-3′, miR-148a-3p: 5′-TCAGTGCACTACAGAACTTTGT-3′). The primers used in PCR are shown in Table 2.

**Table 2:** Primers used for qRT-PCR.

**mRNA**	**Forward primer**	**Reverse primer**
GAPDH	5′-GTGTTCCTACCCCCAATGTG-3′	5′-CATCGAAGGTGGAAGAGTGG-3′
IL-1β	5′-TCCTGTGTAATGAAAGACGGC-3′	5′-TGCTTGTGAGGTGCTGATGTA-3′
IL-6	5′-GGGACTGATGCTGGTGACAA-3′	5′-TCCACGATTTCCCAGAGAACA-3′
IL-10	5′-GCCAGAGCCACATGCTCCTA-3′	5′-GATAAGGCTTGGCAACCCAAGTAA-3′
TNF-α	5′-GAACTGGCAGAAGAGGCACT-3′	5′-CATAGAACTGATGAGAGGGAGG-3′
Notch1	5′-TGACAACTCCTACCTCTGCTTATG-3′	5′-GGTTCACAGGCACATTCGTA-3′
Notch2	5′-GTGTGACATTCCAGGACGCTT-3′	5′-AGTGAAGTCGCCAGTCTGAC-3′

### Western Blotting

Cells and EVs were lysed by cold RIPA lysis buffer (supplemented with PMSF) and then centrifuged at 12,000 rpm at 4°C for 15 min. The supernatant was collected and transferred into a 5-ml tube and then boiled in 100°C water for 10 min. BCA Kit was used for protein quantification. To extract protein from nucleus or cytoplasm separately, cells were lysed using Nuclear and Cytoplasmic Protein Extraction Kit (Sangon Biotech, China) following the manufacturer's instructions, and then the levels of specific proteins were detected. In brief, equal amounts of protein were separated by SDS-PAGE and transferred onto a PVDF membrane. Membranes were blocked with 5% nonfat milk in TBST for 2 h at room temperature and then incubated specifically with primary rabbit monoclonal antibodies against CD9 (1:1,000, Bioworid, US), CD63 (1:1,000, SAB, US), CD81 (1:1,000, Epitomics, US), Alix (1:1,000, Abcam, UK), GAPDH (CST, US), NF-κB p65 (1:1,000, Abcam, US), IKKα/β (1:1,000, Abcam, US), p-IKKα/β (Ser176/180) (1:1,000, CST, US), β-actin (1:1,000, CST, US), and Histone-H3 (1:1,000, Proteintech, China) overnight at 4°C. The next day, the membranes were washed three times for 5 min each with TBST and then incubated with goat anti-rabbit HRP-conjugated IgG secondary antibodies (1:3,000, Boster, Wuhan, China) at 37°C for 1 h. Protein bands were detected using ECL reagent (Millipore, USA) on a FluorChem FC system (Alpha Innotech).

### Hematoxylin and Eosin (H&E) Staining

Tissues were fixed in 10% formalin, dehydrated in alcohol, embedded in paraffin, and then cut into 5 μm-thick sections. The sections were deparaffinized and stained with hematoxylin and eosin (H&E). Images were taken on an FSX100 microscope (Olympus, Japan).

### ELISA

Blood was collected from the left ventricle of the mice and coagulated at room temperature for 30 min. Then, the sample was centrifuged for 15 min at 3,000 rpm, and the serum was collected. The cytokines, including IL-1β, TNF-α, and IL-6, were examined by using ELISAs (Nanjing Jiancheng, China) according to the manufacturer's instructions.

### Statistical Analysis

Data was shown as the mean ± SD. Comparisons between two groups were determined using Mann–Whitney *U* tests; one-way analysis of variance (ANOVA) was applied for multiple group comparisons. Data were analyzed with the SPSS 18.0 program, and *p* < 0.05 was considered a statistically significant difference.

## Results

### Characterization of ADSC-EVs

**Figure 1:**
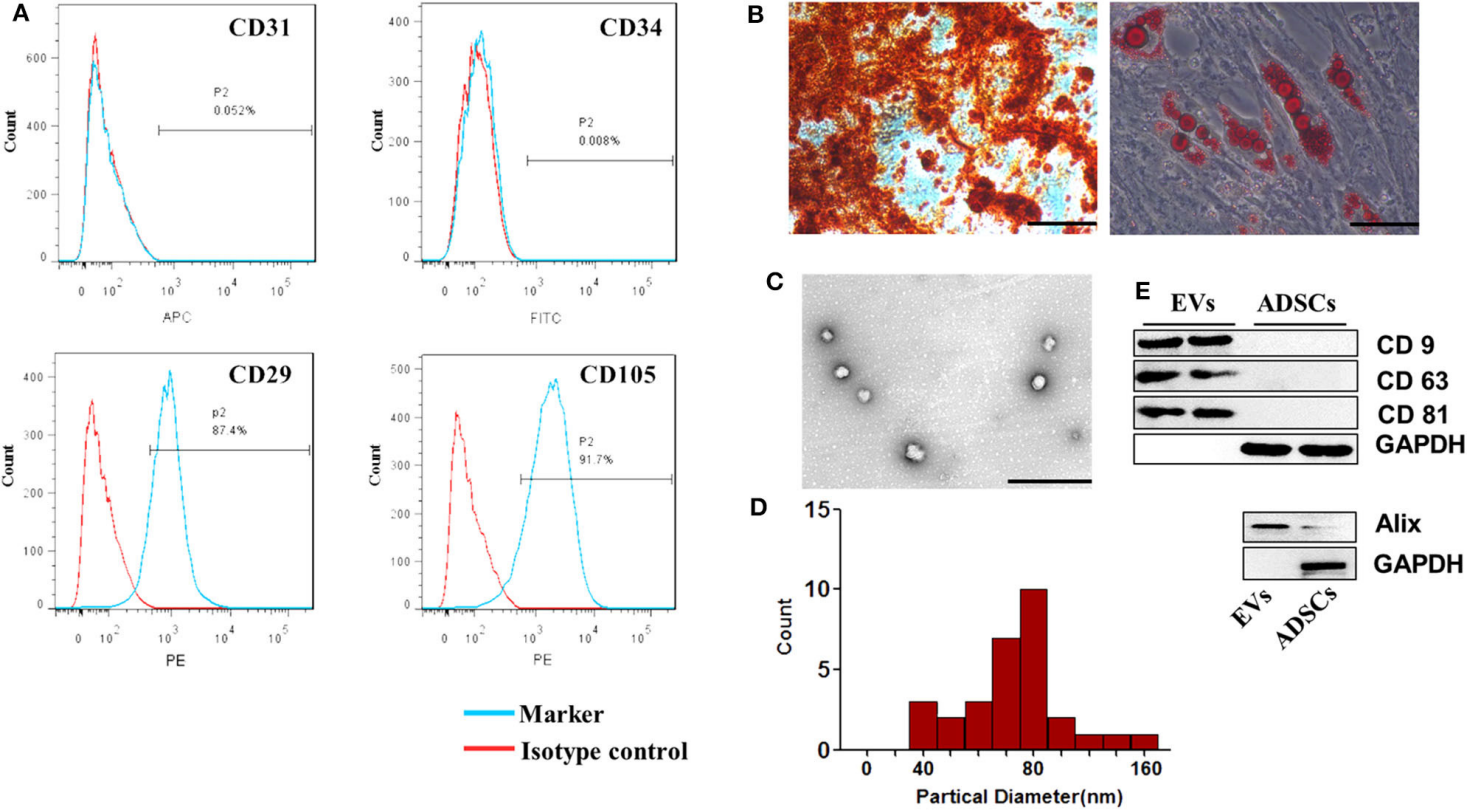
Characterization of ADSCs. **(A)** Flow cytometry analysis showed that these cells were highly positive for CD29 and CD105 but negative for CD31 and CD34 compared with isotype control. **(B)** Representative photographs of lipid droplets stained with Oil red O and calcium deposits stained with Alizarin Red S. Scale bar = 100 μm. **(C)** The morphology of the ADSC-EVs was detected by TEM. Scale bar= 1 μm. **(D)** The diameter distribution of EVs (nm). Four fields were chosen randomly, eight vesicles in each field were randomly chosen to measure the size of the ADSC-EVs. **(E)** CD9, CD63, CD81 and Alix expression in EVs was confirmed by western blotting. *n* = 4.

We first identified the characteristics of ADSC-EVs. ADSCs were identified using flow cytometry assay. The cells were positive for CD29 and CD105 and negative for CD31 and CD34 (Figure 1A). Adipogenic differentiation was demonstrated by Oil red O staining, and osteogenic differentiation was evaluated by Alizarin Red S staining (Figure 1B). Then, EVs isolated from the supernatants of ADSCs were identified. Transmission electron microscope (TEM) showed that the EVs had a typical cup-shaped characteristic morphology. The diameter of the ADSC-EVs was 73.65 ± 25.12 nm, directly measured using the Zeta View system (Figures 1C,D), which was consistent with previous reports. The expression of surface markers CD9, CD63, and CD81 as well as cytosolic protein Alix were estimated by western blotting (Figure 1E). These results showed that ADSC-EVs were isolated successfully.

### ADSC-EVs Inhibited LPS Induced Expression of Inflammatory Factors in Macrophages

**Figure 2:**
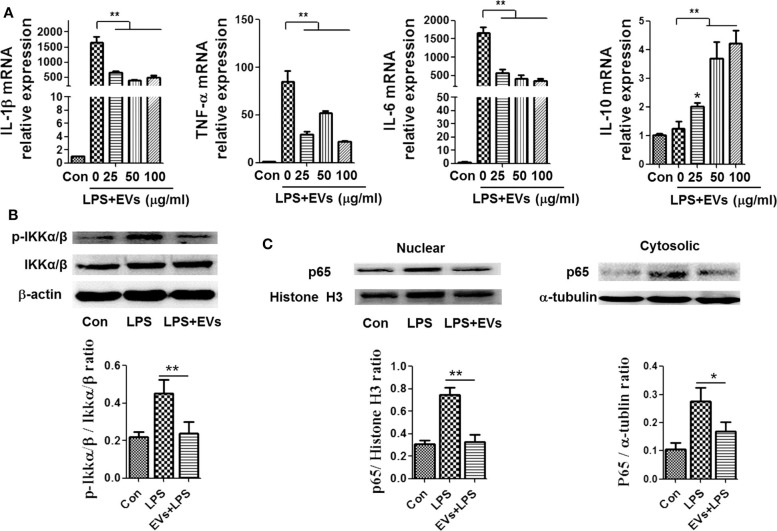
ADSC-EVs treatment alleviated inflammation induced by LPS in macrophages. **(A)** Macrophages were treated with LPS (1 μg/ml) and ADSC-EVs in different concentration gradients, and then, the mRNA levels of IL-1β, TNF-α, IL-6, and IL-10 were assessed by RT-PCR. **(B,C)** Macrophages were treated with LPS (1 μg/ml) or LPS + 25 μg/ml ADSC-EVs, and then, the protein levels of *p-*IKKα/β, as well as p65 in the nucleus and cytosol, were revealed separately by western blotting. *p < 0.05, **p < 0.01 compared with LPS group; *n* = 6.

To explore the effect of ADSC-EVs in inflammation, we used LPS-stimulated macrophages as an inflammatory cell model: LPS (1 μg/ml) stimulation caused a significant increase in the mRNA levels of IL-1β, IL-6 and TNF-α in macrophages (Figure 2A), which suggested that the macrophages were polarized to M1 during inflammation. LPS stimulation resulted in increased levels of proinflammatory cytokines *in vitro*. After administration of different concentrations of ADSC-EVs, the expression of proinflammatory cytokines substantially decreased and the expression of anti-inflammatory cytokine IL-10 increased compared with that in the LPS group (Figure 2A). Similarly, the levels of *p*-IKKα/β proteins were decreased dramatically compared to those of the LPS group (Figure 2B). In the nucleus, p65 was decreased significantly after ADSC-EVs administration (Figure 2C). ADSC-EVs treatment affected the phosphorylation of IKKα/β and the nucleus import of p65 in macrophages. These results suggest that ADSC-EVs inhibited M1 polarization and NF-κB activation induced by LPS.

### ADSC-EVs Decreased the Expression of Proinflammatory Cytokines and Protected Against Organ Injuries in LPS-Injected Mice

**Figure 3:**
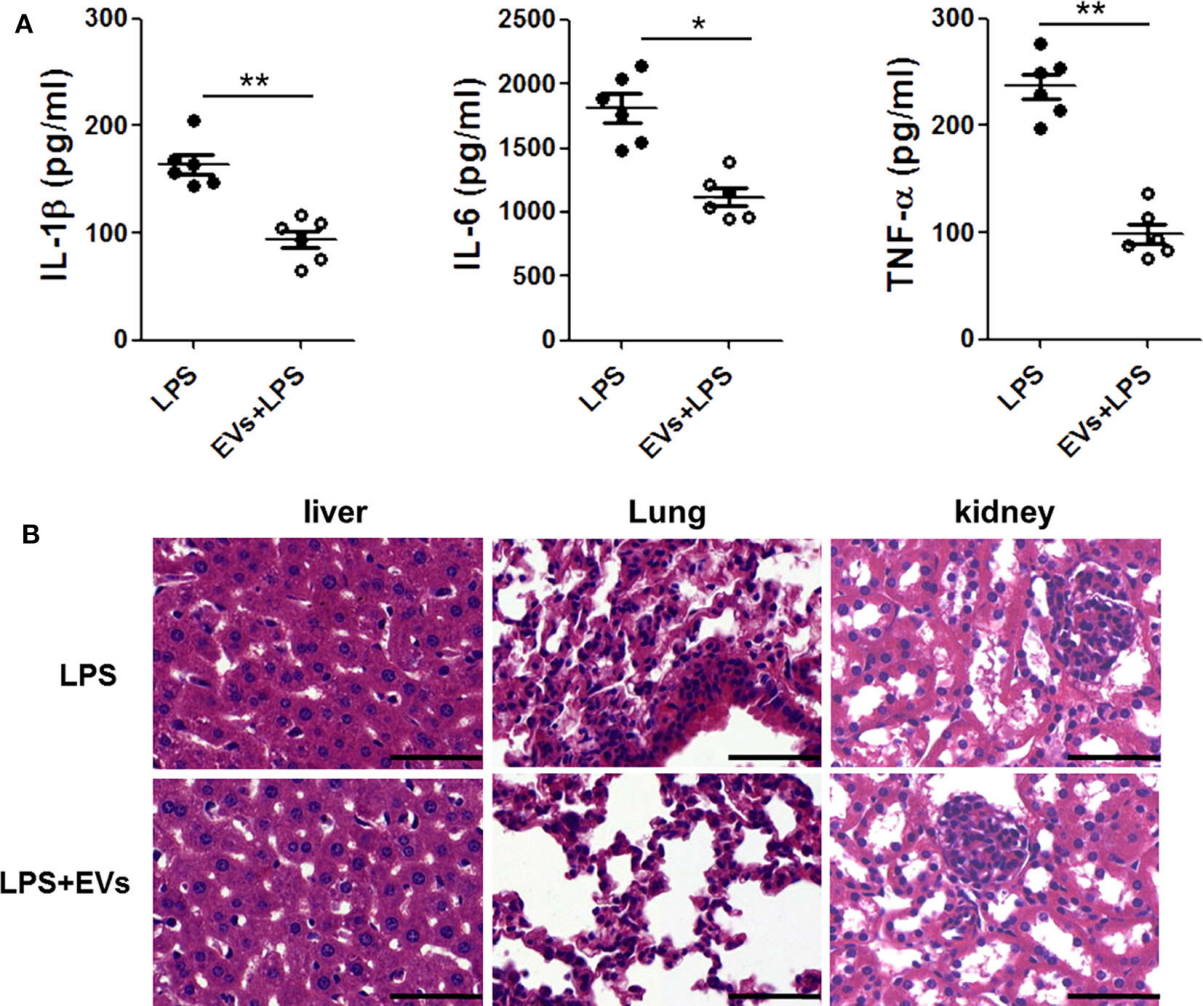
ADSC-EVs alleviated inflammation and organ injury induced by LPS. Mice were treated with LPS (10 mg/kg body weight) or LPS + EVs (200 μg) for 24 h. **(A)** Blood from the left ventricle of the mice was collected, and the serum IL-1β, IL-6, and TNF-α levels were measured using commercial ELISA kits. **(B)** Morphological changes of liver, lung, and kidney tissues from mice after intraperitoneal injection of 10 mg/kg body weight LPS or LPS + 200 μg EVS. Sections were examined by H&E staining and photographed using an FSX100 microscope (Olympus, Japan). *p < 0.05, **p < 0.01 compared with LPS-injected mice; *n* = 6. Scale bar = 50 μm.

To further verify the effect of ADSC-EVs in inflammation, we used LPS-injected (10 mg/kg body weight) mice as an animal model. The proinflammatory cytokines in mice serum were detected by ELISA. Compared with those in LPS group, IL-1β, IL-6 and TNF-α levels were substantially decreased in ADSC-EVs group (Figure 3A).

In Figure 3B, there was significant congestion in vein and the hepatocytes necrosis was visible in LPS group. However, in ADSC-EVs group, hepatocytes necrosis was alleviated, and the congestion was also improved.

In the pulmonary sections, the structures of alveoli were dramatically destroyed in LPS group, and the alveolar exudate was much more severe than that in ADSC-EVs group. There were many infiltrated inflammatory cells in the tissue of LPS group, while these cells were significantly decreased in ADSC-EVs group.

The number of necrotic glomeruli in ADSC-EVs group was much lower than that in LPS group. The morphology of the tubules was almost normal in the ADSC-EVs group, while casts were observed in the tubules of LPS group (Figure 3B).

### ADSC-EVs Alleviated LPS-Induced Notch Signaling Activation in Macrophages

**Figure 4:**
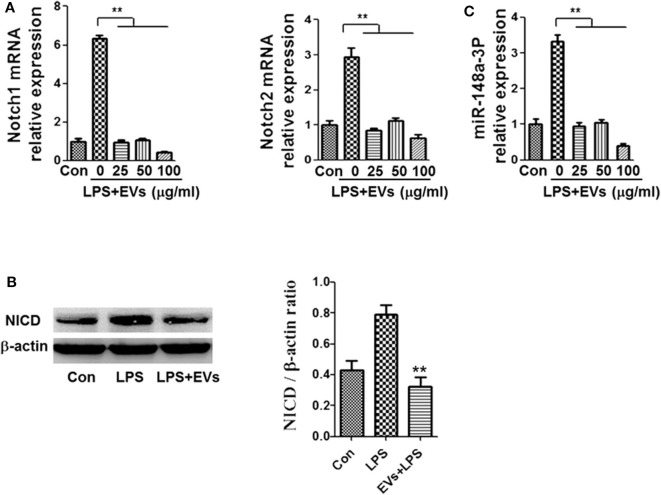
ADSC-EVs treatment led to reduced Notch signaling and miR-148a-3p expression in macrophages. **(A)** Macrophages were treated with LPS (1 μg/ml) and ADSC-EVs at different concentrations, and then, the mRNA levels of Notch1 and Notch2 were assessed by RT-PCR. **(B)** The protein levels of NICD were revealed by western blotting. **(C)** Levels of miR-148a-3p in LPS-treated macrophages with ADSC-EVs administration in different concentration. **p < 0.01; *n* = 6.

To identify the possible mechanisms by which ADSC-EVs alleviated inflammation, we used LPS-induced macrophages as an inflammatory cell model. Notch is one of the most important signaling pathways during inflammation and sepsis (23), so we tried to investigated whether Notch participated in this process. Macrophages were stimulated with LPS (1 μg/ml). Then, the mRNA levels of Notch1 and Notch2 were detected and found that the level of them increased (Figure 4A). After LPS stimulation, the protein level of NICD was also substantially increased in macrophages (Figure 4B). As shown in Figure 4A, treatment with ADSC-EVs induced a significant decrease of Notch1 and Notch2 mRNA levels compared to those of the LPS group (*p* < 0.01). Similarly, the expression levels of NICD proteins were decreased dramatically compared to those in the LPS group (Figure 4B). Since it has been reported that the miRNA profiles of Notch-inactivated and control macrophages showed significantly decreased miR-148a-3p expression in *RBP-J*-deficient macrophages (24), we examined whether miR-148a-3p participated in ADSC-EVs-mediated inhibition of inflammation. As shown in Figure 4C, the expression of miR-148a-3p was also decreased significantly after ADSC-EVs administration compared with that of LPS group.

### Notch Inactivation by *RBP-J* Knockout Alleviated Inflammation Induced by LPS and Suppressed the Activation NF-κB

**Figure 5:**
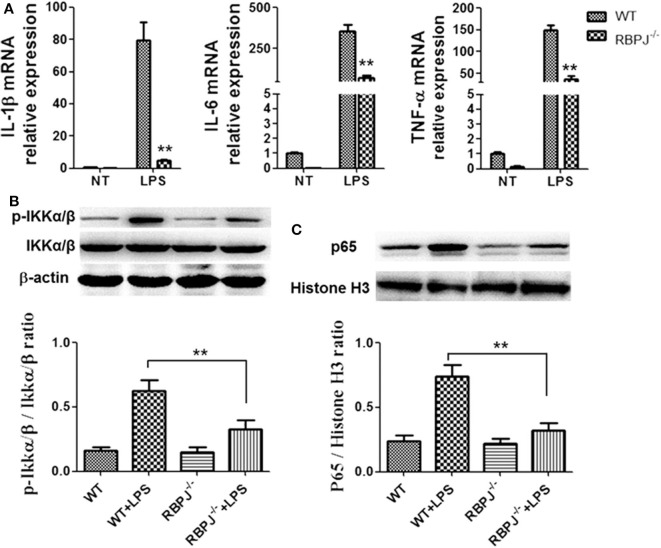
*RBP-J*^−/−^ alleviated LPS induced inflammatory cytokine expression and NF-κB activation. Macrophages from *RBP-J*^−/−^ mice and WT mice were treated with LPS (1 μg/ml). **(A)** The mRNA levels of IL-1β, IL-6, and TNF-α were tested by RT-PCR. **(B,C)** The phosphorylation of IKKα/β and nucleus import of p65 were tested by western blotting. **p < 0.01; *n* = 6.

Then, macrophages from *RBP-J* knockout mice were used. Cells were treated with LPS or control (PBS). Notch inactivation alleviated LPS induced inflammation and M1 polarization. The levels of IL-1β, IL-6 and TNF-α were lower in *RBP-J*^−/−^ macrophages than in normal macrophages (Figure 5A). Then, the phosphorylation of IKKα/β and the nucleus import of p65 were also assessed by western blotting. *RBP-J* knockout led to decreased activation of NF-κB (Figures 5B,C).

### The Inflammation in *RBP-J^−/−^* Mice Was Milder Than That in WT Mice After LPS Stimulation

**Figure 6:**
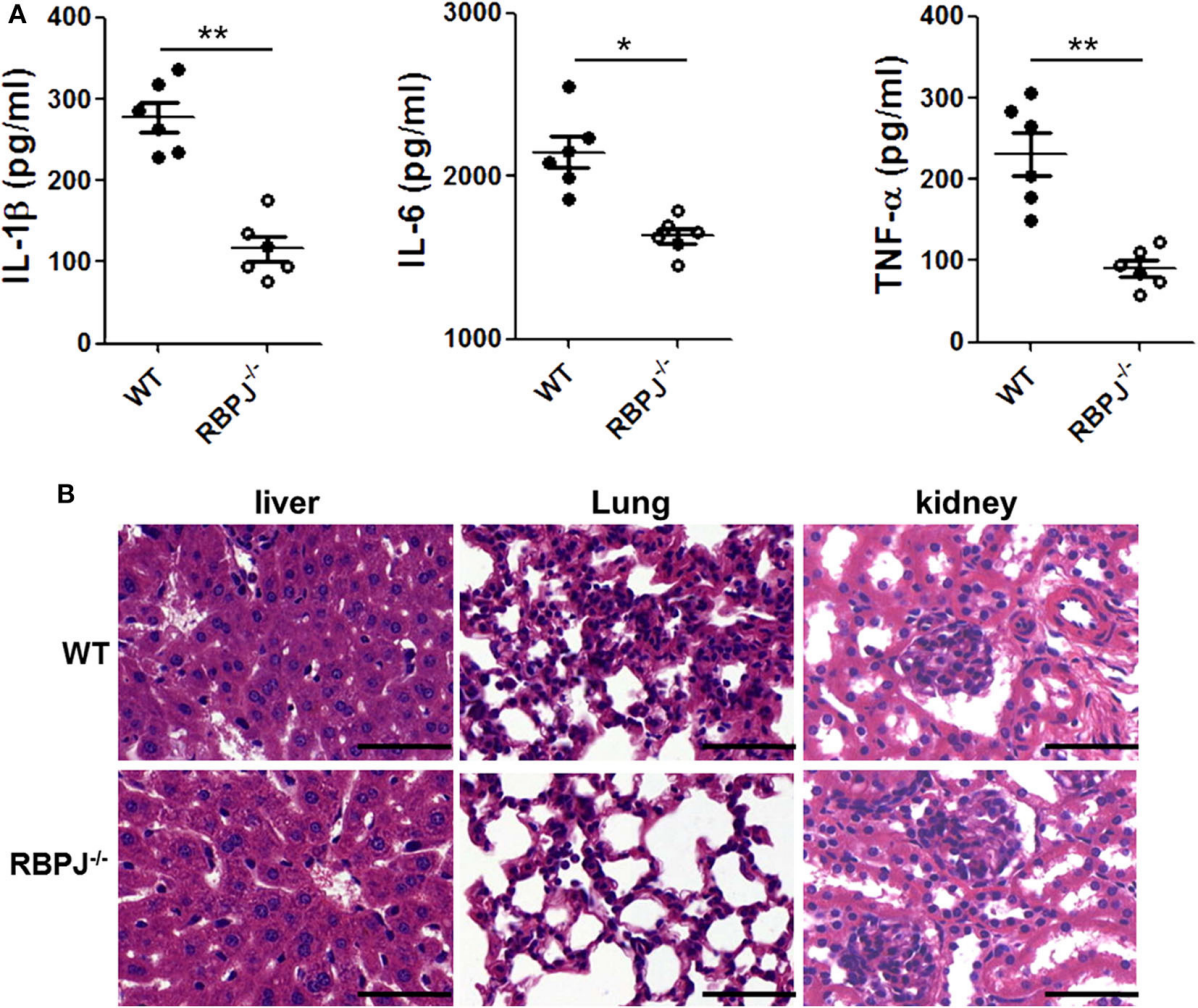
*RBP-J*^−/−^ alleviated inflammation and organ injury induced by LPS *in vivo*. *RBP-J*^−/−^ mice and WT mice were treated with LPS (10 mg/kg body weight) for 24 h. **(A)** Blood from the left ventricle of mice was collected, and serum IL-1β, IL-6, and TNF-α levels were measured using commercial ELISA kits. **(B)** Morphological changes of liver, lung, and kidney tissues from mice after intraperitoneal injection of 10 mg/kg body weight LPS. Sections were examined by H&E staining and photographed using an FSX100 microscope (Olympus, Japan). *p < 0.05, **p < 0.01 compared with LPS-injected mice; *n* = 6. Scale bar = 50 μm.

Then, we verified the effect of Notch signaling *in vivo*. Twenty-four hours after LPS injection, the proinflammatory cytokines in mice serum were detected by ELISA. Compared with WT mice, IL-1β, IL-6 and TNF-α levels were substantially decreased in *RBP-J*^−/−^ mice (Figure 6A). In Figure 6B, the integrity of the organ structures shown in H&E staining was better in *RBP-J*^−/−^ mice than in WT mice.

### MicroRNA-148a-3p Was Located Downstream of the Notch Signal

**Figure 7:**
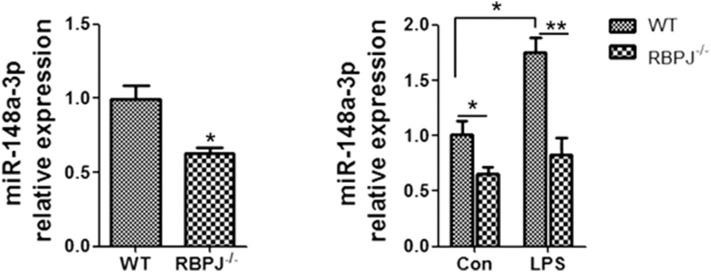
RT-PCR showed the level of miR-148a-3p in LPS-treated (1 μg/ml) macrophages from WT mice or *RBP-J*^−/−^ mice. *p < 0.05, **p < 0.01 compared with LPS group; *n* = 6.

Since miR-148a-3p was related to Notch signaling, we tried to confirm the position of miR-148a-3p. The level of miR-148a-3p was significantly lower in *RBP-J*^−/−^ macrophages than the WT macrophages. With LPS stimulation, the level of miR-148a-3p was merely increased in *RBP-J*^−/−^ macrophages (Figure 7).

### miR-148a-3p Promoted M1 Macrophage Polarization and Enhanced Inflammation

**Figure 8:**
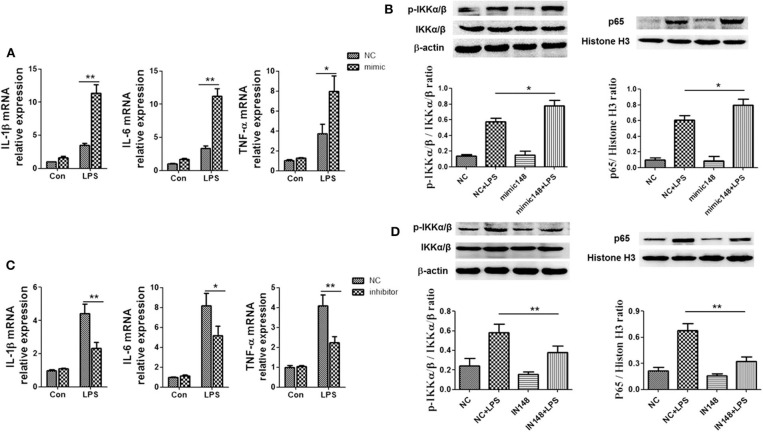
miR-148a-3p could promote inflammation and M1 polarization. **(A,B)** Macrophages were treated with miR-148a-3p mimic or NC, and then, PBS or LPS (1 μg/ml) was added. The mRNA levels of IL-1β, TNF-α, and IL-6 were determined by RT-PCR. The protein level of *p-*IKKα/β in cell and p65 in nucleus were detected by western blotting. **(C,D)** Macrophages were treated with miR-148a-3p inhibitor or NC, and then, and then, PBS or LPS (1 μg/ml) was added. The mRNA levels of IL-1β, TNF-α and IL-6 were determined by RT-PCR and the protein levels of *p-*IKKα/β and p65 in nucleus were revealed by western blotting. *p < 0.05, **p < 0.01; *n* = 6.

To verify whether miR-148a-3p could regulate M1 macrophage polarization, we used miR-148a-3p mimic or inhibitor to enhance or suppress the expression of miR-148a-3p. The results showed that the mRNA levels of IL-1β, TNF-α and IL-6 were upregulated in miR-148a-3p mimic group and the protein level of *p-*IKKα/β in cell and p65 in nucleus were increased compared with NC group (Figures 8A,B). Conversely, when macrophages were stimulated with miR-148a-3p inhibitor, the expression levels of IL-1β, TNF-α, and IL-6 decreased as well as the protein levels of *p-*IKKα/β and nucleus import of p65 were decreased dramatically when miR-148a-3p was inhibited (Figures 8C,D).

### ADSC-EVs Could Alleviate Inflammation and Organ Injury in Mice via miR-148a-3p

**Figure 9:**
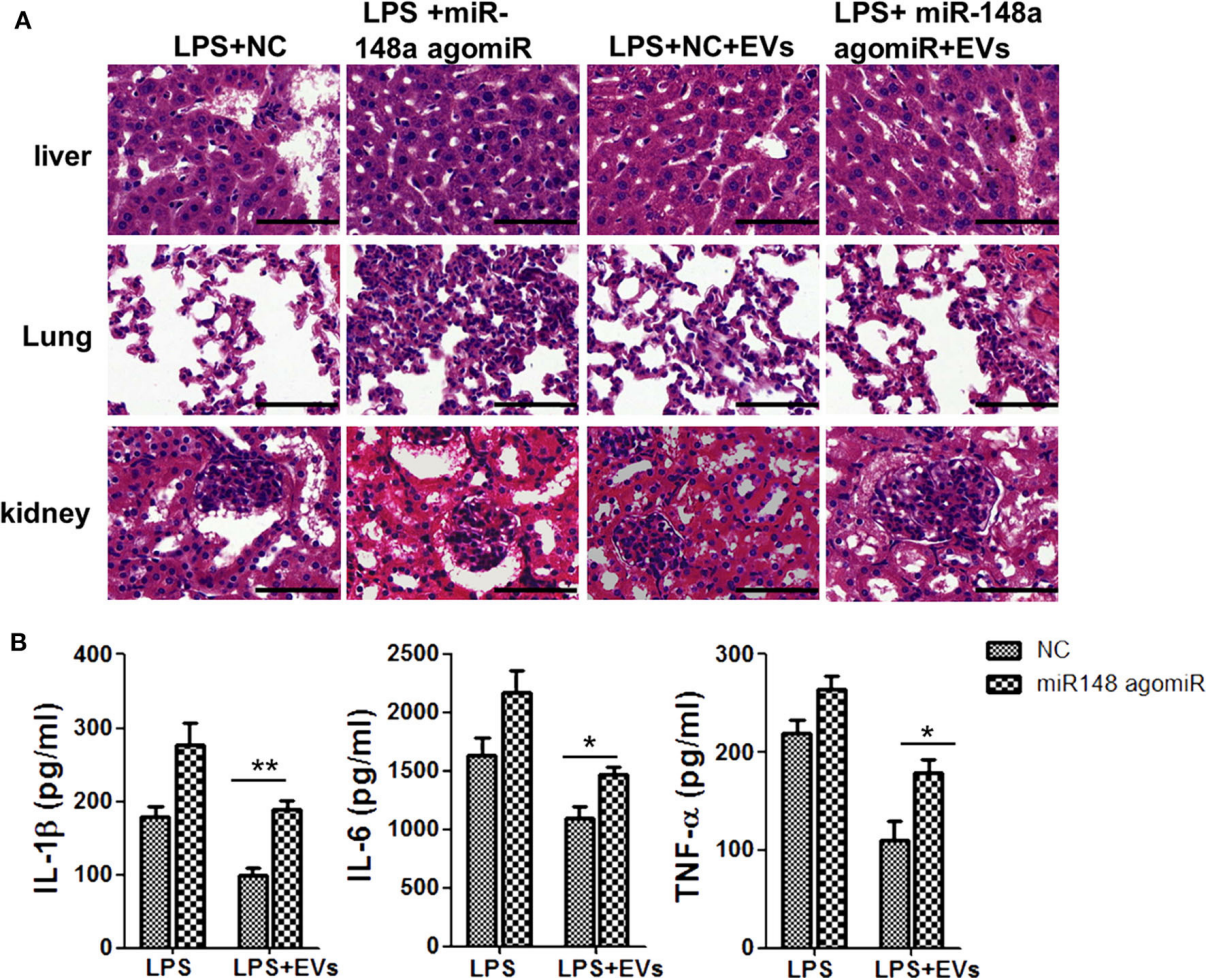
miR-148a-3p agomiR diminished the protective effect of ADSC-EVs in LPS induced sepsis. Mice were treated with LPS (10 mg/kg body weight) and then divided into four groups as NC group, miR-148a-3p agomiR group, NC +EVs group and miR-148a-3p agomiR + EVs group. **(A)** The morphological changes of liver, lung, and kidney tissues in NC group, NC + EVs group, miR-148a-3p agomiR group and miR-148a-3p agomiR + EVs group were shown by H&E staining. Sections were observed and photographed using an FSX100 microscope (Olympus, Japan). **(B)** The protein levels of IL-1β and TNF-α in these four groups of mice were determined by ELISA. *p < 0.05, **p < 0.01; *n* = 6. Scale bar = 50 μm.

Further, to explore whether the protective effects of ADSC-EVs were related to the decrease of miR-148a-3p, we first injected mice with LPS and then divided them into four groups: NC group, miR-148a-3p agomiR group, NC +EVs group and miR-148a-3p agomiR + EVs group. H&E staining showed that organ injury was obviously aggravated in miR-148a-3p agomiR group compared to NC group. With the administration of ADSC-Evs, organ injury was alleviated, while with the administration of miR-148a-3p agomiR, the protective effect of ADSC-EVs was diminished (Figure 9A). ADSC-EVs administration downregulated the expression of IL-1β, IL-6, and TNF-α compared with that of NC group, while this effect was also eliminated by the miR-148a-3p agomiR (Figure 9B).

## Discussion

An inappropriate immune response may lead to uncontrolled inflammation and organ injury (25, 26). In the clinic, diagnosis of sepsis in the early stage and reversal of sepsis following multiple organ injuries are a major challenge (27). Mesenchymal stem cells (MSCs) can differentiate into different types of cells, including osteoblasts, chondroblasts, and adipocytes (28). MSCs can be acquired from diverse tissues, such as bone marrow, umbilical cord blood, placental tissue, and adipose tissue (29). Numerous studies have confirmed that MSCs could be effective in various inflammatory diseases, such as inflammatory bowel disease, trauma, and sepsis (30, 31). The therapeutic potential of MSCs in inflammatory disorders is immeasurable. Adipose tissue-derived stem cells (ADSCs) are commonly researched since they can be easily acquired from adipose tissue. Several studies have shown that ADSCs have anti-inflammatory, anti-aging, injury repair and immunomodulatory effects by paracrine secretion of various cytokines, angiogenic stimulators and apoptosis factors (32).

Extracellular vesicles (EVs) are vesicles of varying sizes released from cells through both the endosomal pathway and by budding from the plasma membrane, including exosomes, microvesicles, microparticles, and oncosomes. EVs can be released by MSCs and then influence adaptive and innate immunity through exchange of contents among different types of immune cells (33). It has been demonstrated that ADSCs and ADSC-derived exosomes could suppress inflammation (34–36). However, little is known about the effects of ADSCs in inflammation. In this study, we discussed the potential protective effects of ADSC-derived EVs in inflammation during sepsis.

We first identified ADSC and ADSC-EVs and then investigated the effectiveness of ADSC-EVs therapy in LPS-induced inflammation both *in vitro* and *in vivo*. It was found that ADSC-EVs could attenuate inflammation, which was related to Notch signaling and miR-148a-3p. Given the importance of macrophages in immune system and inflammation, macrophages were used as the cell model. Research shows that ADSCs could alleviate inflammation by regulating M1 to M2 macrophage polarization (37, 38). However, the exact mechanism by which ADSCs regulate the differentiation process of macrophages is unclear. ADSC-EVs at different concentrations were given to macrophages stimulated by LPS. The inflammatory cytokines (IL-1β, IL-6, and TNF-α) substantially decreased after the administration of ADSC-EVs compared with those only stimulated with LPS. The phosphorylation of IKKα/β and p65 in nucleus were dramatically downregulated after the administration of ADSC-EVs (Figure 2), indicating that the NF-κB signal, which is related to inflammation and tissue necrosis, was inhibited by the administration of ADSC-EVs.

A high incidence of pulmonary disorder is observed in sepsis (39, 40), followed by dysfunction of the liver, intestine, kidney, hematological system and cardiovascular system (41). To verify the anti-inflammatory properties of ADSC-EVs *in vivo*, we used a sepsis mice model. The results were in accordance with the *in vitro* study. ADSC-EVs decreased the expression of inflammatory cytokines in mice and protected against organ injuries after LPS injection (Figure 3). This result suggested that ADSC-EVs could improve the prognosis of severe inflammation.

Then, we tried to explore the possible mechanisms *in vitro*. Previous studies have shown that Notch signaling is closely related to inflammation and immune function (23). It has been reported that Notch signaling could activate NF-κB signaling in macrophages, by which the balance of inflammation is affected (42). In rheumatoid arthritis patients, Notch signaling is significantly activated in macrophages compared with that in osteoarthritis patients (43). In this study, LPS was used to stimulate macrophages, and we found that the expression of proinflammatory cytokines increased significantly, accompanied by the elevation of Notch signaling molecules (Figures 4A,B). The use of ADSC-EVs suppressed Notch signaling and then inhibited the activation of NF-κB pathway, thereby reducing the inflammatory response. During this process, miR-148a-3p was also inhibited. Various miRNAs participate in the host response during sepsis (44, 45). Several studies have shown that various miRNAs are involved in macrophage differentiation; that is, miRNAs can promote M1 macrophage polarization and suppress M2 macrophage phenotype (46, 47). Huang et al. reported that miR-148a-3p was related to Notch signaling in macrophages (48), suggesting that Notch signaling promotes monocyte differentiation and M1 macrophage activation through miR-148a-3p. We confirmed that ADSC-EVs could alleviate inflammation in mice through the regulation of Notch/miR-148a-3p, and with the treatment of ADSC-EVs, the level of PTEN in LPS induced macrophages increased (Supplementary Figure 1C). MiRs could exert different functions through different targets. MiR-148a-3p has over 800 target genes in human beings. Since Huang et al. reported that PTEN was one of the target genes of miR-148a-3p, we also tested the expression of PTEN and found that after treatment with ADSC-EVs, the level of PTEN increased. Kim et al. (49) reported that NRP1 was also one of the targets of miR-148a/b-3p, which mediated antiangiogenic pathways and was important for overcoming endothelial cell dysfunction and angiogenic disorders.

To explore the specific link between Notch signaling and M1/M2 macrophage polarization, *RBP-J*^−/−^ mice were used. It was found that in *RBP-J*^−/−^ macrophages, the inflammation induced by LPS was milder than that in the WT macrophages, indicating that M1 polarization was partially inhibited (Figure 5). Level of PTEN also increased in *RBP-J*^−/−^ mice (Supplementary Figure 1D). The *in vivo* study also confirmed this conclusion (Figure 6). However, it is still important to illustrate the relationship among Notch signaling, miR-148a-3p and inflammation. Therefore, miR-148 inhibitor and miR-148 mimics were used. It was found that the level of miR-148a-3p decreased in *RBP-J*^−/−^ macrophages. When *RBP-J*^−/−^ macrophages were stimulated with LPS, the level of miR-148a-3p was upregulated less than that in normal macrophages (Figure 7). We concluded that Notch was upstream of miR-148a-3p. The overexpression of miR-148a-3p could lead to aggravated inflammation, while the inhibition of miR-148a-3p could alleviate inflammation. The overexpression of miR-148a-3p led to the M1 polarization of macrophages and the activation of NF-κB signaling (Figure 9).

**Figure 10:**
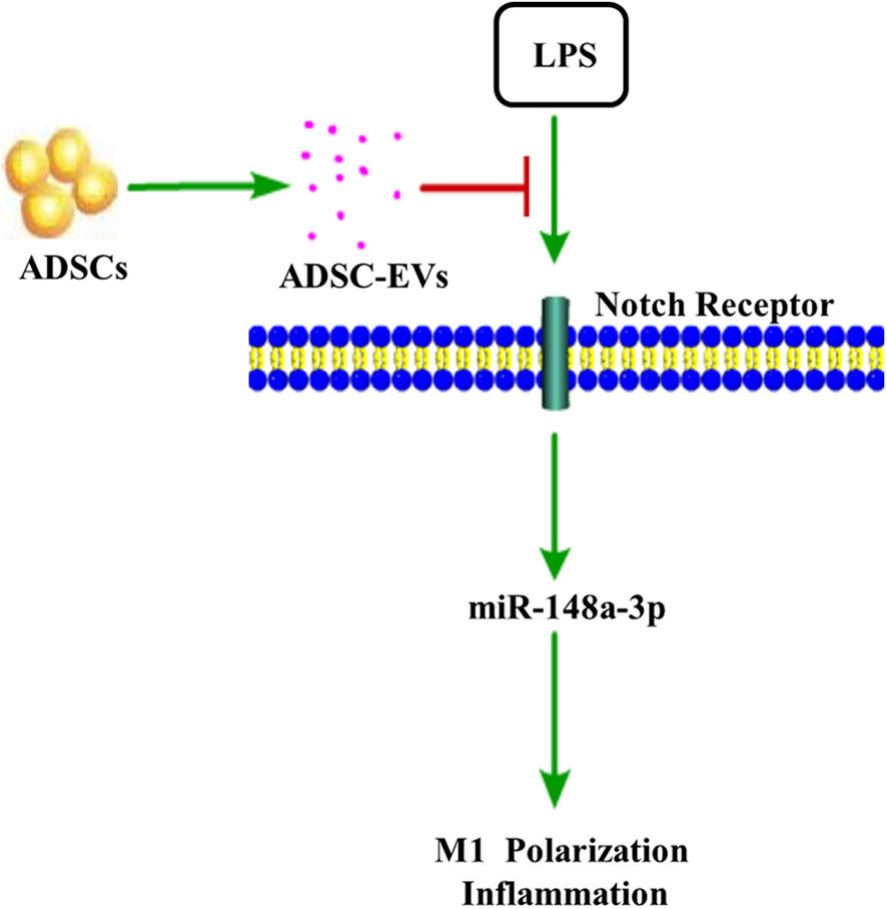
ADSC-EVs treatment attenuated inflammation induced by lipopolysaccharide (LPS). In LPS-induced inflammation, Notch signaling was activated. ADSC-EVs suppressed LPS induced inflammation through Notch/miR-148a-3p signaling, which could also affect macrophage polarization.

In conclusion, we confirmed that ADSC-EVs could have a positive impact on inflammation induced by LPS by suppressing Notch signaling. MiR-148a-3p was one of the important factors that affected Notch signaling to regulate inflammation (Figure 10).

## Data Availability Statement

The datasets generated for this study are available on request to the corresponding author.

## Ethics Statement

The animal study was reviewed and approved by Ethics Committee of Xijing Hospital affiliated with the Fourth Military Medical University.

## Author Contributions

DH and SX designed the project. ML, YL, and MC performed most of the experiments. XB and JL collected and analyzed data. XB and LL wrote the manuscript. JL and KT built the animal model. JL, DH, and SX revised the manuscript and prepared for the submission. All authors contributed to the article and approved the submitted version.

## Conflict of Interest

The authors declare that the research was conducted in the absence of any commercial or financial relationships that could be construed as a potential conflict of interest.

## References

[1] Singer M Deutschman CS Seymour CW Shankar-Hari M Annane D Bauer M. The Third International Consensus Definitions for Sepsis and Septic Shock (Sepsis-3). JAMA. (2016) 315:801–10. 10.1001/jama.2016.0287 26903338 PMC4968574

[2] Seymour CW Kennedy JN Wang S Chang CH Elliott CF Xu Z. Derivation, validation, and potential treatment implications of novel clinical phenotypes for sepsis. JAMA. (2019) 321:2003–17. 10.1001/jama.2019.5791 31104070 PMC6537818

[3] Jekarl DW Kim KS Lee S Kim M Kim Y. Cytokine and molecular networks in sepsis cases: a network biology approach. Eur Cytokine Netw. (2018) 29:103–11. 10.1684/ecn.2018.0414 30547887

[4] Jiang C Li Z Quan H Xiao L Zhao J Jiang C. Osteoimmunology in orthodontic tooth movement. Oral Dis. (2015) 21:694–704. 10.1111/odi.12273 25040955

[5] Luan HH Wang A Hilliard BK Carvalho F Rosen CE Ahasic AM. GDF15 Is an inflammation-induced central mediator of tissue tolerance. Cell. (2019) 178:1231–44 e11. 10.1016/j.cell.2019.07.033 31402172 PMC6863354

[6] Orecchioni M Ghosheh Y Pramod AB Ley K. Macrophage polarization: different gene signatures in M1(LPS+) vs. classically and M2(LPS-) vs. alternatively activated macrophages. Front Immunol. (2019) 10:1084. 10.3389/fimmu.2019.01084 31178859 PMC6543837

[7] Drevets DA Canono BP Campbell PA. Measurement of bacterial ingestion and killing by macrophages. Curr Protoc Immunol. (2015) 109:14 6 1–17. 10.1002/0471142735.im1406s109 25845563

[8] Alcayaga-Miranda F Cuenca J Martin A Contreras L Figueroa FE Khoury M. Combination therapy of menstrual derived mesenchymal stem cells and antibiotics ameliorates survival in sepsis. Stem Cell Res Ther. (2015) 6:199. 10.1186/s13287-015-0192-0 26474552 PMC4609164

[9] Pedrazza L Cubillos-Rojas M de Mesquita FC Luft C Cunha AA Rosa JL. Mesenchymal stem cells decrease lung inflammation during sepsis, acting through inhibition of the MAPK pathway. Stem Cell Res Ther. (2017) 8:289. 10.1186/s13287-017-0734-8 29273091 PMC5741936

[10] Johnson CL Soeder Y Dahlke MH. Concise review: mesenchymal stromal cell-based approaches for the treatment of acute respiratory distress and sepsis syndromes. Stem Cells Transl Med. (2017) 6:1141–51. 10.1002/sctm.16-0415 28186706 PMC5442840

[11] Hu Y Li G Zhang Y Liu N Zhang P Pan C. Upregulated TSG-6 expression in ADSCs inhibits the BV2 microglia-mediated inflammatory response. Biomed Res Int. (2018) 2018:7239181. 10.1155/2018/7239181 30584538 PMC6280241

[12] Shen T Zheng QQ Shen J Li QS Song XH Luo HB. Effects of adipose-derived mesenchymal stem cell exosomes on corneal stromal fibroblast viability and extracellular matrix synthesis. Chin Med J. (2018) 131:704–12. 10.4103/0366-6999.226889 29521294 PMC5865317

[13] Kocan B Maziarz A Tabarkiewicz J Ochiya T Banas-Zabczyk A. Trophic activity and phenotype of adipose tissue-derived mesenchymal stem cells as a background of their regenerative potential. Stem Cells Int. (2017) 2017:1653254. 10.1155/2017/1653254 28757877 PMC5516761

[14] Melo SA Sugimoto H O'Connell JT Kato N Villanueva A Vidal A. Cancer exosomes perform cell-independent microRNA biogenesis and promote tumorigenesis. Cancer Cell. (2014) 26:707–21. 10.1016/j.ccell.2014.09.005 25446899 PMC4254633

[15] Wang Y Yu D Liu Z Zhou F Dai J Wu B. Exosomes from embryonic mesenchymal stem cells alleviate osteoarthritis through balancing synthesis and degradation of cartilage extracellular matrix. Stem Cell Res Ther. (2017) 8:189. 10.1186/s13287-017-0632-0 28807034 PMC5556343

[16] Rashed MH Bayraktar E Helal GK Abd-Ellah MF Amero P Chavez-Reyes A. Exosomes: from garbage bins to promising therapeutic targets. Int J Mol Sci. (2017) 18:538. 10.3390/ijms18030538 28257101 PMC5372554

[17] Schorey JS Cheng Y Singh PP Smith VL. Exosomes and other extracellular vesicles in host-pathogen interactions. EMBO Rep. (2015) 16:24–43. 10.15252/embr.201439363 25488940 PMC4304727

[18] Lakkaraju A Rodriguez-Boulan E. Itinerant exosomes: emerging roles in cell and tissue polarity. Trends Cell Biol. (2008) 18:199–209. 10.1016/j.tcb.2008.03.002 18396047 PMC3754907

[19] Liu J Jiang M Deng S Lu J Huang H Zhang Y. miR-93-5p-containing exosomes treatment attenuates acute myocardial infarction-induced myocardial damage. Mol Ther Nucleic Acids. (2018) 11:103–15. 10.1016/j.omtn.2018.01.010 29858047 PMC5852413

[20] Zhang W Bai X Zhao B Li Y Zhang Y Li Z. Cell-free therapy based on adipose tissue stem cell-derived exosomes promotes wound healing via the PI3K/Akt signaling pathway. Exp Cell Res. (2018) 370:333–42. 10.1016/j.yexcr.2018.06.035 29964051

[21] Li P Kaslan M Lee SH Yao J Gao Z. Progress in exosome isolation techniques. Theranostics. (2017) 7:789–804. 10.7150/thno.18133 28255367 PMC5327650

[22] Feito MJ Diez-Orejas R Cicuendez M Casarrubios L Rojo JM Portoles MT. Characterization of M1 and M2 polarization phenotypes in peritoneal macrophages after treatment with graphene oxide nanosheets. Colloids Surf B Biointerfaces. (2019) 176:96–105. 10.1016/j.colsurfb.2018.12.063 30594708

[23] Shang Y Smith S Hu X. Role of Notch signaling in regulating innate immunity and inflammation in health and disease. Protein Cell. (2016) 7:159–74. 10.1007/s13238-016-0250-0 26936847 PMC4791423

[24] Zhao JL Huang F He F Gao CC Liang SQ Ma PF. Forced activation of notch in macrophages represses tumor growth by upregulating miR-125a and disabling tumor-associated macrophages. Cancer Res. (2016) 76:1403–15. 10.1158/0008-5472.CAN-15-2019 26759236

[25] Seymour CW Liu VX Iwashyna TJ Brunkhorst FM Rea TD Scherag A. Assessment of clinical criteria for sepsis: for the third international consensus definitions for sepsis and septic shock (sepsis-3). JAMA. (2016) 315:762–74. 10.1001/jama.2016.0288 26903335 PMC5433435

[26] Wang L Li Y Wang X Wang P Essandoh K Cui S. GDF3 protects mice against sepsis-induced cardiac dysfunction and mortality by suppression of macrophage pro-inflammatory phenotype. Cells. (2020) 9. 10.3390/cells9010120 31947892 PMC7017037

[27] Jacob JA. New sepsis diagnostic guidelines shift focus to organ dysfunction. JAMA. (2016) 315:739–40. 10.1001/jama.2016.0736 26903319

[28] Bernardo ME Fibbe WE. Mesenchymal stromal cells: sensors and switchers of inflammation. Cell Stem Cell. (2013) 13:392–402. 10.1016/j.stem.2013.09.006 24094322

[29] Hu X Garcia M Weng L Jung X Murakami JL Kumar B. Identification of a common mesenchymal stromal progenitor for the adult haematopoietic niche. Nat Commun. (2016) 7:13095. 10.1038/ncomms13095 27721421 PMC5062560

[30] Mao F Kang JJ Cai X Ding NF Wu YB Yan YM. Crosstalk between mesenchymal stem cells and macrophages in inflammatory bowel disease and associated colorectal cancer. Contemp Oncol. (2017) 21:91–7. 10.5114/wo.2017.68616 28947877 PMC5611497

[31] Matthay MA Pati S Lee JW. Concise review: mesenchymal stem (stromal) cells: biology and preclinical evidence for therapeutic potential for organ dysfunction following trauma or sepsis. Stem Cells. (2017) 35:316–24. 10.1002/stem.2551 27888550

[32] Bertolini F Lohsiriwat V Petit JY Kolonin MG. Adipose tissue cells, lipotransfer and cancer: a challenge for scientists, oncologists and surgeons. Biochim Biophys Acta. (2012) 1826:209–14. 10.1016/j.bbcan.2012.04.004 22546620

[33] Maas SLN Breakefield XO Weaver AM. Extracellular vesicles: unique intercellular delivery vehicles. Trends Cell Biol. (2017) 27:172–88. 10.1016/j.tcb.2016.11.003 27979573 PMC5318253

[34] Shang Q Bai Y Wang G Song Q Guo C Zhang L. Delivery of adipose-derived stem cells attenuates adipose tissue inflammation and insulin resistance in obese mice through remodeling macrophage phenotypes. Stem Cells Dev. (2015) 24:2052–64. 10.1089/scd.2014.0557 25923535

[35] Kotani T Masutani R Suzuka T Oda K Makino S Ii M. Anti-inflammatory and anti-fibrotic effects of intravenous adipose-derived stem cell transplantation in a mouse model of bleomycin-induced interstitial pneumonia. Sci Rep. (2017) 7:14608. 10.1038/s41598-017-15022-3 29097816 PMC5668313

[36] Zhao H Shang Q Pan Z Bai Y Li Z Zhang H. Exosomes from adipose-derived stem cells attenuate adipose inflammation and obesity through polarizing m2 macrophages and beiging in white adipose tissue. Diabetes. (2018) 67:235–47. 10.2337/db17-0356 29133512

[37] Xie J Jones TJ Feng D Cook TG Jester AA Yi R. Human adipose-derived stem cells suppress elastase-induced murine abdominal aortic inflammation and aneurysm expansion through paracrine factors. Cell Transplant. (2017) 26:173–89. 10.3727/096368916X692212 27436185 PMC5657756

[38] Pilny E Smolarczyk R Jarosz-Biej M Hadyk A Skorupa A Ciszek M. Human ADSC xenograft through IL-6 secretion activates M2 macrophages responsible for the repair of damaged muscle tissue. Stem Cell Res Ther. (2019) 10:93. 10.1186/s13287-019-1188-y 30867059 PMC6417195

[39] Sun Y Sun L Liu S Song J Cheng J Liu J. Effect of emodin on Aquaporin 5 expression in rats with sepsis-induced acute lung injury. J Tradit Chin Med. (2015) 35:679–84. 10.1016/S0254-6272(15)30159-X 26742314

[40] Inagawa R Okada H Takemura G Suzuki K Takada C Yano H. Ultrastructural alteration of pulmonary capillary endothelial glycocalyx during endotoxemia. Chest. (2018) 154:317–25. 10.1016/j.chest.2018.03.003 29555594

[41] Xiu G Sun J Li X Jin H Zhu Y Zhou X. The role of HMGB1 in BMSC transplantation for treating MODS in rats. Cell Tissue Res. (2018) 373:395–406. 10.1007/s00441-018-2823-0 29637307

[42] Ventura S Aryee DN Felicetti F De Feo A Mancarella C Manara MC. CD99 regulates neural differentiation of Ewing sarcoma cells through miR-34a-Notch-mediated control of NF-kappaB signaling. Oncogene. (2016) 35:3944–54. 10.1038/onc.2015.463 26616853 PMC4967355

[43] Ogando J Tardaguila M Diaz-Alderete A Usategui A Miranda-Ramos V Martinez-Herrera DJ. Notch-regulated miR-223 targets the aryl hydrocarbon receptor pathway and increases cytokine production in macrophages from rheumatoid arthritis patients. Sci Rep. (2016) 6:20223. 10.1038/srep20223 26838552 PMC4738320

[44] O'Connell RM Rao DS Baltimore D. microRNA regulation of inflammatory responses. Annu Rev Immunol. (2012) 30:295–312. 10.1146/annurev-immunol-020711-075013 22224773

[45] Wu X Yang J Yu L Long D. Plasma miRNA-223 correlates with risk, inflammatory markers as well as prognosis in sepsis patients. Medicine. (2018) 97:e11352. 10.1097/MD.0000000000011352 29979415 PMC6076081

[46] Li H Jiang T Li MQ Zheng XL Zhao GJ. Transcriptional regulation of macrophages polarization by microRNAs. Front Immunol. (2018) 9:1175. 10.3389/fimmu.2018.01175 29892301 PMC5985397

[47] Fortunato O Borzi C Milione M Centonze G Conte D Boeri M. Circulating mir-320a promotes immunosuppressive macrophages M2 phenotype associated with lung cancer risk. Int J Cancer. (2019) 144:2746–61. 10.1002/ijc.31988 30426475 PMC6590261

[48] Huang F Zhao JL Wang L Gao CC Liang SQ An DJ. miR-148a-3p mediates notch signaling to promote the differentiation and M1 activation of macrophages. Front Immunol. (2017) 8:1327. 10.3389/fimmu.2017.01327 29085372 PMC5650608

[49] Kim H Ko Y Park H Zhang H Jeong Y Kim Y. MicroRNA-148a/b-3p regulates angiogenesis by targeting neuropilin-1 in endothelial cells. Exp Mol Med. (2019) 51:1–11. 10.1038/s12276-019-0344-x 31723119 PMC6853980

