# Supplementary material for: Extracellular Vesicles From Adipose Tissue-Derived Stem Cells Affect Notch-miR148a-3p Axis to Regulate Polarization of Macrophages and Alleviate Sepsis in Mice
Source: Front Immunol. 2020 Jul 3;11:1391. doi: 10.3389/fimmu.2020.01391 (PMC7347748; doi:10.3389/fimmu.2020.01391)
Supplement: Supplementary file 1 [file Data_Sheet_1.DOCX]

**Supplementary Material**

**
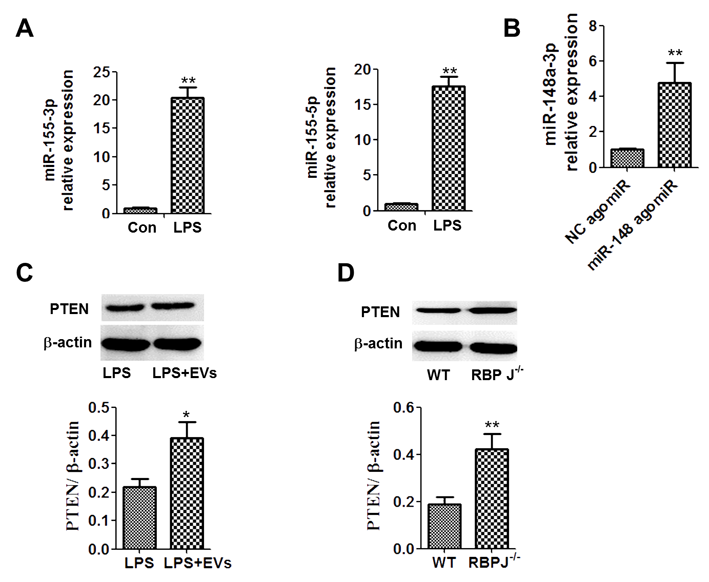
**

**Supplementary Figure 1**. **(A)**. Macrophages were treated with LPS (1μg/ml) for 4 h and the expression of miR-155-3p and miR-155-5p were detected by RT-PCR. **(B)**. Mice were injected with miR-148a-3p agomiR or NC agomiR from tail vein on 3 consecutive days (12.1 μmol/kg/day). The level of miR-148a-3p in the pulmonary of mice were detected 12 h after the last injection of miR-148a-3p agomiR. **(C).** Macrophages were treated with LPS (1μg/ml) or LPS (1μg/ml) + EVs (25μg/ml). The expression of PTEN were detected 24 h by western blotting. **(D).** The expression of PTEN in *RBP-J*^-/-^ macrophage and WT macrophage. **p* < 0.05; ***p* < 0.01; n = 6.
